# The international Perinatal Outcomes in the Pandemic (iPOP) study: protocol

**DOI:** 10.12688/wellcomeopenres.16507.1

**Published:** 2021-02-02

**Authors:** Sarah J. Stock, Helga Zoega, Meredith Brockway, Rachel H. Mulholland, Jessica E. Miller, Jasper V. Been, Rachael Wood, Ishaya I. Abok, Belal Alshaikh, Adejumoke I. Ayede, Fabiana Bacchini, Zulfiqar A. Bhutta, Bronwyn K. Brew, Jeffrey Brook, Clara Calvert, Marsha Campbell-Yeo, Deborah Chan, James Chirombo, Kristin L. Connor, Mandy Daly, Kristjana Einarsdóttir, Ilaria Fantasia, Meredith Franklin, Abigail Fraser, Siri Eldevik Håberg, Lisa Hui, Luis Huicho, Maria C. Magnus, Andrew D. Morris, Livia Nagy-Bonnard, Natasha Nassar, Sylvester Dodzi Nyadanu, Dedeke Iyabode Olabisi, Kirsten R. Palmer, Lars Henning Pedersen, Gavin Pereira, Amy Racine-Poon, Manon Ranger, Tonia Rihs, Christoph Saner, Aziz Sheikh, Emma M. Swift, Lloyd Tooke, Marcelo L. Urquia, Clare Whitehead, Christopher Yilgwan, Natalie Rodriguez, David Burgner, Meghan B. Azad, iPOP Study Team

**Affiliations:** 1 Usher Institute, University of Edinburgh, Edinburgh, UK; 2 Centre for Big Data Research in Health, Faculty of Medicine, UNSW Sydney, Sydney, Australia; 3 Centre of Public Health Sciences, Faculty of Medicine, University of Iceland, Reykjavík, Iceland; 4 Pediatrics and Child Health, University of Manitoba, Winnipeg, Canada; 5 Infection and Immunity, Murdoch Children’s Research Institute, Royal Children’s Hospital, Parkville, Australia; 6 Division of Neonatology, Department of Paediatrics, Erasmus MC - Sophia Children’s Hospital, University Medical Centre Rotterdam, Rotterdam, The Netherlands; 7 Department of Obstetrics and Gynecology, Erasmus MC, University Medical Centre Rotterdam, Rotterdam, The Netherlands; 8 Department of Public Health, University Medical Centre Rotterdam, Rotterdam, The Netherlands; 9 Public Health Scotland, Edinburgh, UK; 10 Centre for Clinical Brain Sciences, University of Edinburgh, Edinburgh, UK; 11 Department of Paediatrics, University of Jos, Jos, Nigeria; 12 Department of Pediatrics, University of Calgary, Calgary, Canada; 13 Department of Paediatrics, College of Medicine, University of Ibadan, Ibadan, Nigeria; 14 University College Hospital, Ibadan, Nigeria; 15 Canadian Premature Babies Foundation, Toronoto, Canada; 16 Center of Excellence in Women Child Health, The Aga Khan University South-Central Asia & East Africa, Karachi, Pakistan; 17 Department of Medical Epidemiology and Biostatistics, Karolinska Institute, Stockholm, Sweden; 18 National Perinatal Epidemiology and Statistics Unit, Centre for Big Data Research in Health, UNSW Sydney, Sydney, Australia; 19 Dalla Lana School of Public Health, University of Toronto, Toronto, Canada; 20 Department of Chemical Engineering and Applied Chemistry, University of Toronto, Toronto, Canada; 21 Centre for Global Health, Usher Institute, University of Edinburgh, Edinburgh, UK; 22 Dalhousie University, Halifax, Canada; 23 Malawi-Liverpool-Wellcome Clinical Research Programme, Blantyre, Malawi; 24 Department of Health Sciences, Carleton University, Ottawa, Canada; 25 IWK Health Centre, Halifax, Canada; 26 Advocacy & Policymaking, Irish Neonatal Health Alliance, Dublin, Ireland; 27 Unit of Fetal Medicine and Prenatal Diagnosis Institute for Maternal and Child Health, IRCCS Burlo Garofolo, Trieste, Italy; 28 Division of Biostatistics, Department of Preventive Medicine, Keck School of Medicine, University of Southern California, Los Angeles, USA; 29 MRC Integrative Epidemiology Unit,, University of Bristol, Bristol, UK; 30 Population Health Sciences, Bristol Medical School, Bristol, UK; 31 Centre for Fertility and Health, Norwegian Institute of Public Health, Oslo, Norway; 32 Department of Obstetrics and Gynaecology, University of Melbourne, Melbourne, Australia; 33 Centro de Investigación en Salud Materna e Infantil, Universidad Peruana Cayetano Heredia, Lima, Peru; 34 School of Medicine, Universidad Peruana Cayetano Heredia, Lima, Peru; 35 Centro de Investigación para el Desarrollo Integral y Sostenible, Universidad Peruana Cayetano Heredia, Lima, Peru; 36 Health Data Research UK, London, UK; 37 Melletted a helyem Egyesulet, Budapest, Hungary; 38 Children’s Hospital at Westmead Clinical School, University of Sydney, Sydney, Australia; 39 School of Public Health, Curtin University, Perth, Australia; 40 Education, Culture, and Health Opportunities (ECHO) Research Group International, Aflao, Ghana; 41 Department of Pediatrics, Federal Medical Centre, Abeokuta, Nigeria; 42 Monash Health Department of Obstetrics & Gynaecology, Monash University, Clayton, Australia; 43 Department of Obstetrics & Gynaecology, Aarhus University Hospital, Aarhus, Denmark; 44 Clinical Medicine & Biomedicine, Aarhus University, Aarhus, Denmark; 45 Telethon Kids Institute, Nedlands, Australia; 46 Melinda and Bill Gates Foundation, Seattle, USA; 47 BC Children’s & Women’s Hospital Research Institute, School of Nursing, University of British Columbia, Vanvouver, Canada; 48 Federal Statistical Office, Neuchatel, Switzerland; 49 Department of Pediatric Endocrinology, Diabetology, and Metabolism, University Children`s Hospital Bern, Inselspital, Bern, Switzerland; 50 Department of Midwifery, Faculty of Nursing, University of Iceland, Reykjavík, Iceland; 51 Department of Neonatology, University of Cape Town, Cape Town, South Africa; 52 Department of Neonatology, Groote Schuur Hospital, Cape Town, South Africa; 53 Manitoba Centre for Health Policy, Department of Community Health Sciences, Rady Faculty of Health Sciences, University of Manitoba, Winnipeg, Canada; 54 Pregnancy Research Centre, The Royal Women's Hospital, Melbourne, Australia; 55 Department of Paediatrics, University of Melbourne, Parkville, VIC, Australia; 56 Children’s Hospital Research Institute of Manitoba, The Children’s Hospital Foundation of Manitoba, Winnipeg, Canada

**Keywords:** pandemic lockdowns, COVID-19, preterm birth, stillbirth, low birth weight, perinatal outcomes, global trends

## Abstract

Preterm birth is the leading cause of infant death worldwide, but the causes of preterm birth are largely unknown. During the early COVID-19 lockdowns, dramatic reductions in preterm birth were reported; however, these trends may be offset by increases in stillbirth rates. It is important to study these trends globally as the pandemic continues, and to understand the underlying cause(s). Lockdowns have dramatically impacted maternal workload, access to healthcare, hygiene practices, and air pollution - all of which could impact perinatal outcomes and might affect pregnant women differently in different regions of the world.

In the international Perinatal Outcomes in the Pandemic (iPOP) Study, we will seize the unique opportunity offered by the COVID-19 pandemic to answer urgent questions about perinatal health. In the first two study phases, we will use population-based aggregate data and standardized outcome definitions to: 1) Determine rates of preterm birth, low birth weight, and stillbirth and describe changes during lockdowns; and assess if these changes are consistent globally, or differ by region and income setting, 2) Determine if the magnitude of changes in adverse perinatal outcomes during lockdown are modified by regional differences in COVID-19 infection rates, lockdown stringency, adherence to lockdown measures, air quality, or other social and economic markers, obtained from publicly available datasets. We will undertake an interrupted time series analysis covering births from January 2015 through July 2020.

The iPOP Study will involve at least 121 researchers in 37 countries, including obstetricians, neonatologists, epidemiologists, public health researchers, environmental scientists, and policymakers. We will leverage the most disruptive and widespread “natural experiment” of our lifetime to make rapid discoveries about preterm birth. Whether the COVID-19 pandemic is worsening or unexpectedly improving perinatal outcomes, our research will provide critical new information to shape prenatal care strategies throughout (and well beyond) the pandemic.

## Introduction

The COVID-19 pandemic and response measures taken to mitigate the spread of infection have dramatically impacted health and health systems across the globe. Maternal and child health is at high risk, especially in low- and middle-income countries where resources for health care are already limited ^1^. Pandemic response measures may have profound societal impacts owing to the combination of constrained supply, reduced resources, suppressed human interaction, and worsening socio-economic inequality. Projections already suggest about a 45% increase in child deaths and 39% increase in maternal deaths across low- and middle-income countries related to the pandemic ^2^.

Unexpectedly, recent evidence from some high-income countries suggests unprecedented reductions in preterm births (up to 90% in Denmark and 23% in the Netherlands) and births classified as very low birth weight (70% in Ireland) following the COVID-19 lockdowns ^3– 5^. At the same time, reports from Nepal and India show an alarming increase in stillbirths and preterm births related to changes in maternity care ^6, 7^. Increases in stillbirth have also been seen in the UK and Italy (Lazio region) ^8, 9^. In California, preterm birth rates seem largely unchanged during the pandemic period, except for a modest increase (11%) in very preterm birth, driven primarily by the Hispanic/Latinx population ^10^.

It is critical to evaluate these seemingly contrasting trends and to understand the underlying mechanisms. The pandemic mitigation measures have substantially impacted maternal workload ^11^, access to healthcare ^12^, hygiene practices ^13^, air pollution ^14^, nutrition ^15– 17^, and non-SARS-CoV-2 infection ^18^, each of which may have affected maternal and perinatal outcomes disproportionately in different socio-economic and regional settings. It is plausible that changes in exposures to inflammatory triggers, such as infections ^19– 21^ and air pollution ^22^, may be partly responsible for changes to some perinatal outcomes, such as spontaneous preterm birth. We will therefore seize the unique opportunity resulting from the global COVID-19 pandemic to answer pressing questions on pandemic lockdowns and perinatal health on a global scale.

To address the impact of the pandemic response measures on perinatal health, the international Perinatal Outcomes in the Pandemic (iPOP) study, is working in partnership with the International COVID-19 Data Alliance (ICODA, supported by the COVID-19 Therapeutics Accelerator) to establish an inclusive international research programme that will collaborate to focus on key questions relevant to countries across the globe of all income levels. The initial focus of iPOP will be on the impact of COVID‐19 pandemic lockdowns on perinatal outcomes, including preterm birth, low birth weight, and stillbirth.

### Objective

The overall objective of iPOP is to determine the impact of pandemic lockdowns on perinatal outcomes worldwide, and to investigate potential mechanisms underlying these effects.

## Protocol

### Study goals and conceptual framework

The overarching goal of iPOP is to:

•    Investigate the impact of COVID-19 lockdowns on perinatal outcomes (including preterm birth, low birth weight, and stillbirth);

•    Compare the impact of COVID-19 lockdown on perinatal outcomes by country income setting: low-income countries (LICs), lower-middle-income countries (LMICs), upper-middle-income countries (UMICs) and high-income countries (HICs);

•    Explore the underlying societal and etiological factors that are associated with between-country differences in the impact of COVID-19 lockdown on perinatal outcomes.

**Figure 1.**
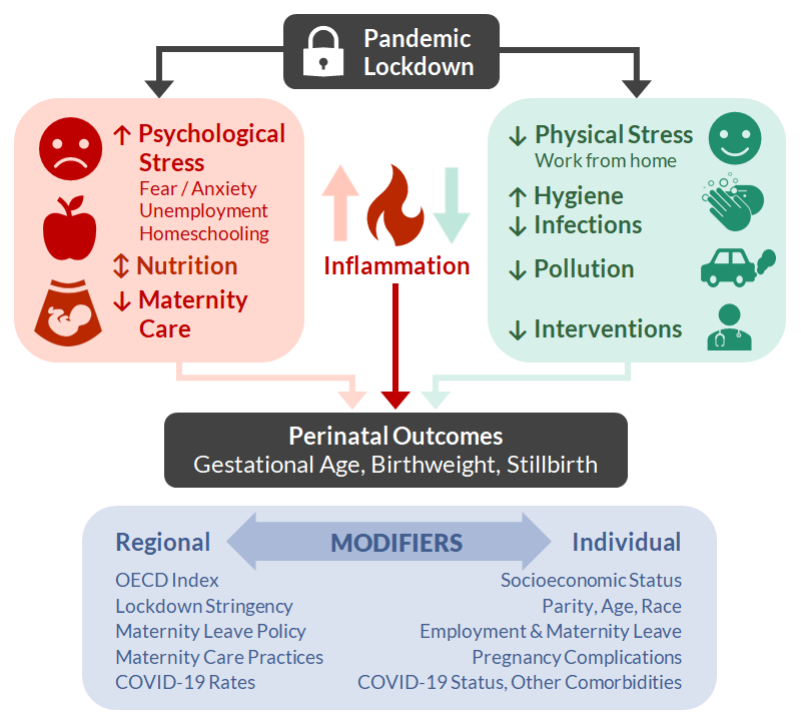
iPOP Study Conceptual Framework. OECD, Organisation for Economic Co-operation and Development.

The conceptual framework ( Figure 1) for the iPOP project is intended to help build a series of work packages (WPs), each increasing in complexity and building on the previous findings. Within this protocol we address WP1 and WP2.

*WP1 - Describe global trends and regional differences in adverse perinatal outcomes during COVID-19 pandemic lockdowns, using population-based aggregate data and standardized outcome definitions:* Report rates of preterm birth, low birth weight, and stillbirth and describe changes during the pandemic lockdown. Determine if these changes are consistent globally, or if they differ between or within LIC, LMIC, UMIC and HIC settings.

*WP2 - Address contextual influences and mechanisms for changes in preterm birth, stillbirth, and low birth weight during the COVID-19 pandemic, using population-based aggregate and publicly available data*: determine if the magnitude of regional changes in adverse perinatal outcomes during lockdown are potentially modified by regional differences in COVID-19 infection rates, lockdown stringency, adherence to lockdown measures, air quality, and other social and economic markers available from public datasets.

**Figure 2.**
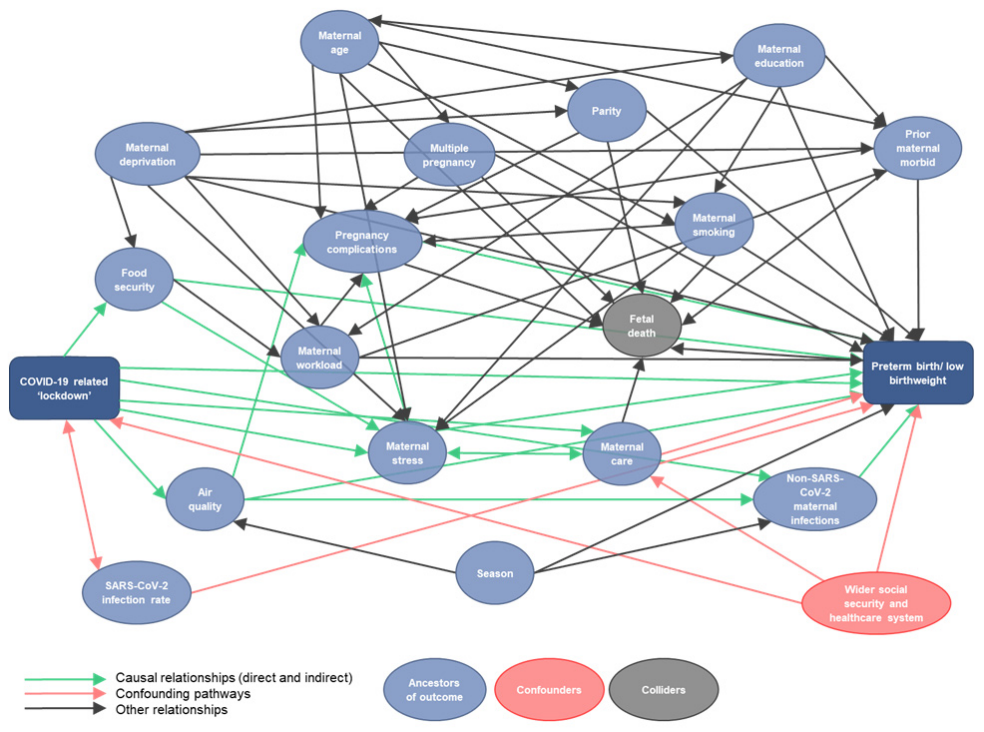
Full directed acyclic graph (DAG) of lockdown to perinatal outcomes.

Possible mechanisms driving the association between pandemic lockdown measures and perinatal outcomes are represented in a directed acyclic graph (DAG) in Figure 2.

### Aims

In WP1, we will estimate the impact of pandemic lockdowns on global incidence of preterm birth, low birth weight, and stillbirth using population-based data. Specifically, we will answer the following questions:

 1. Has implementation of COVID-19 pandemic lockdowns been associated with a change in preterm birth rate (<37 weeks gestation)?

 2. Does the association vary:

a) When the outcome is restricted to *spontaneous preterm birth* (preterm birth preceded by spontaneous contractions and/or preterm prelabour rupture of membranes)?
b) When the outcome is restricted to *early preterm birth* (<32 weeks gestation)?

3. Does any association with preterm birth remain when analyses are restricted to live births only (i.e. exclusion of stillbirths)?

4. Has implementation of COVID-19 pandemic lockdown been associated with a change in low birth weight rate (<2500g)?

5. Has implementation of COVID-19 pandemic lockdown been associated with a change in stillbirth rate?

6. Has implementation of COVID-19 pandemic lockdown been associated with a change in post term birth rate (≥42 weeks gestation)?

7. Do any observed associations with preterm birth, low birth weight, and stillbirth vary by country income setting (LIC, LMIC, UMIC, HIC)?

Our primary hypothesis is that the rate of spontaneous preterm birth, low birth weight and/or stillbirth is changed during pandemic lockdowns worldwide. Our secondary hypothesis is that the magnitude and/or direction of the change in the spontaneous preterm birth, low birth weight, and/or stillbirth varies by country income setting as classified by the World Bank income grouping.

In WP2 we will build directly from WP1 with the addition of national/regional characteristics derived from publicly available datasets to explore the influence of the association between lockdown measures and adverse perinatal outcomes.

Specifically, we will address the following questions:

8. Are the direction and magnitude of any changes in preterm birth, low birth weight and/or stillbirth rates observed in WP1 modified by factors such as:

a. Lockdown stringency index (see section *Exposures* below)
b. Adherence to lockdown indicated by traffic and social mobility data
c. Ambient air quality
d. COVID-19 rates
e. Parental leave policy
f. Socioeconomic setting
g. Gross domestic product
h. World region (East Asia and Pacific, Europe and Central Asia, Latin America & the Caribbean, Middle East and North Africa, North America, South Asia, Sub-Saharan Africa).

### General approach

We will analyse aggregate population-based data provided by collaborators from different national/regional sites. Our primary method of analysis will be an interrupted time series analysis (ITSA) and we will consider alternative quasi-experimental approaches as appropriate.

Results from each contributing site will be meta-analysed, if appropriate. We will classify data into one of three tiers (Standard, Enhanced, or Investigative) based on the nature of the datasets in terms of population coverage, quality and completeness, and availability of required variables ( Table 1). Standard data meet the minimum criteria for inclusion in the main analysis of at least one primary or secondary outcome. Enhanced data meet the minimum criteria for inclusion in the main analysis of a primary or secondary outcome, as well as including additional data allowing inclusion in one or more additional or sensitivity analyses. Investigative data do not meet the minimum criteria to be included in the main analysis of a primary or secondary outcome but can be included in supplementary analyses exploring trends (designed to promote wide geographical coverage).

**Table 1.** Characteristics of datasets included in the iPOP Study.

Coverage	
Standard dataset	National, subnational, population-based data
Enhanced dataset	National, subnational, population-based data
Investigative dataset	Institutional level data or other non-population-based data
Completeness	
Standard dataset	≥90% births with a meaningful/feasible value for an outcome
Enhanced dataset	≥90% births with a meaningful/feasible value for an outcome
Investigative dataset	<90% births with a meaningful/feasible value for an outcome
Time period	
Standard dataset	1 Jan 2015 to 31 July 2020
Enhanced dataset	1 Jan 2015 to most recent data available
Investigative dataset	1 Jan 2018 to 31 July 2020
Breakdown of data	
Standard dataset	By consecutive calendar month
Enhanced dataset	By consecutive calendar month + by consecutive International Standard (ISO) week and
Investigative dataset	By any other time frame or discontinuous data
Birth categories	
Standard dataset	All births
Enhanced dataset	All births +/- Live births and stillbirths +/- Spontaneous preterm births
Investigative dataset	Live births only, population-based data / All births, institutional level data or other non-population-based data
Gestation	
Standard dataset	28 ^+0^ - 36 ^+6^ weeks ≥37 ^+0^ weeks
Enhanced dataset	22 ^+0^ - 27 ^+6^ weeks 28 ^+0^ - 31 ^+6^ weeks 32 ^+0^ - 36 ^+6^ weeks 37 ^+0^ - 41 ^+6^ weeks ≥42 ^+0^ weeks
Investigative dataset	Preterm birth identified by checkbox (without registration of gestational age)
Birth weight	
Standard dataset	1000 – 2499g ≥2500g
Enhanced dataset	500 – 999g (if available) 1000 – 1499g 1500 – 2499g ≥2500g
Investigative dataset	Low birth weight identification by checkbox (without registration of birth weight)

A single contributing dataset may be categorised in different tiers for different analyses, e.g. a dataset with low completeness on gestational age but high completeness on birth weight might be categorised as investigative for analyses of preterm birth and as enhanced for analyses of low birth weight.

Wherever possible we have aligned our definitions with those of the World Health Organization (WHO) ^23^. To allow meaningful comparison of international data, in our main analyses definitions we have used a gestational age threshold of 28 weeks, and birth weight threshold or 1000g for inclusion (identified by the extension “_m”). This is in recognition that inclusion of extreme preterm and extremely low birthweight births can disrupt the validity of such comparisons. However, these births will be included in definitions for the enhanced analyses (identified by the extension “_e”).

**Figure 3.**
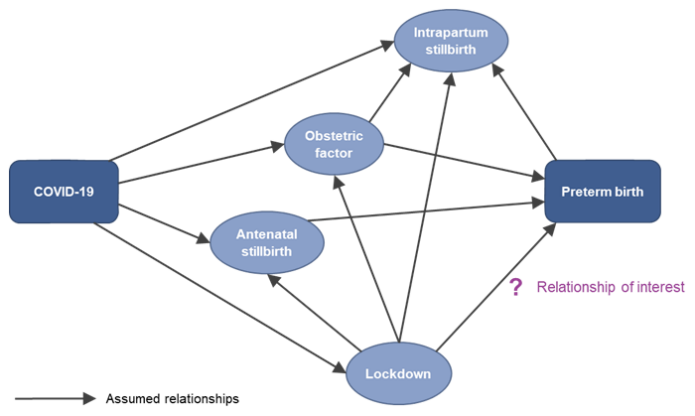
Directed acyclic graph (DAG) - Simple version (work package 1).

We have chosen to use a denominator of total births for our primary outcome of preterm birth. Figure 3 shows a simplified DAG justifying use of this denominator. COVID-19 infection might increase susceptibility to intrapartum stillbirth, whereas lockdown might reduce susceptibility to intrapartum stillbirth (because of fewer infections/ less pollution) or increase it (due to changes in access to maternity care). Intrapartum stillbirth here is a collider so we should not condition on intrapartum stillbirth. As it is difficult to get good information on whether stillbirths are intrapartum (as opposed to antenatal), total birth is appropriate as the denominator.

## Methods

### Study population

Our aim is to capture, at a minimum, data on all births (live and stillbirth) from 28 ^+0^ to 44 ^+6^ weeks gestation inclusive; or above ≥1000g birth weight. We also aim to capture additional data on all births (live and stillbirth) from 22 ^+0^ to 27 ^+6^ weeks gestation, or between 500g and 999g. These data will be included in enhanced analyses.

### Study period

The main analysis study period is January 1, 2015 to July 31, 2020, covering the first lockdown period (in 2020) and the previous five calendar years. We will include data from January 1, 2018 to July 31, 2020 in investigative analysis if earlier data is not available. We will request the most recent data available to allow enhanced analyses covering a wider time period.

### Exposures

The primary exposure will be a binary variable for lockdown based on the stringency index. We will use the stringency index from the Oxford COVID-19 Government Response Tracker. The Oxford COVID-19 Government Response Tracker provides a systematic cross-national, cross-temporal measure to understand how government responses have evolved over the full period of the disease’s spread. It collects information on different policies and interventions that governments have instituted in response to the COVID-19 pandemic and using standardized series of indicators creates a suite of composites indices to measure the extent of these responses. The indicators cover information on containment and closure policies (e.g. school closures and restrictions in movement) (C1-C8); economic policies (e.g. income support to citizens or provision of foreign aid) (E1-E4); and record health system policies (e.g. COVID-19 testing regimes or emergency investments into healthcare) (H1-H5). The lockdown stringency index is calculated using only the policy indicators C1-C8 and H1. The value of the index on any given day is the average of nine sub-indices pertaining to the individual policy indicators, each taking a value between 0 and 100. If the most stringent policy is only present in a limited area or region, a binary flag variable denotes limited scope. The codebook for the stringency index is publicly available.

We will define lockdown as a score of ≥50 on the Oxford COVID-19 Government Response Tracker stringency index. The decision on this arbitrary cut off has been influenced by scoping of stringency index data in high income settings and comparison of stringency indexes in settings which have and have not implemented lockdown measures. For example, Sweden (which has not had a ‘lockdown’) never implemented measures during the study period that added up to higher than 50 on the stringency index, compared to neighbouring Denmark, which scored above 50 throughout the study period in 2020. We will record timing of reaching a score ≥50 separately for each country/region. Our primary analysis will focus on the start date of pandemic lockdown defined as the first date when a country/region’s stringency exceeded 49 (i.e. as a stringency score of ≥50).

Subsequent analyses may include the:

•    Time period of pandemic lockdown: defined as a continuous calendar period during which a country/region has a stringency score of ≥50

•    Total duration of pandemic lockdown: defined as the sum of all calendar periods during which a country/region has a stringency score of ≥50

Note: The beginning and length of lockdown may vary by country/region

### Comparator

Births during the 2020 lockdown periods will be compared with births occurring before the first date when a country/region’s stringency exceeded 49 (i.e. as a stringency score of ≥50), defined by lockdown stringency index in each country/region. The exact comparator time period may vary by country/region.

### Outcomes

Primary outcome

•    Preterm birth rate_m (main analysis: any birth 28 ^+0^- 36 ^+6^ weeks gestation; denominator total births ≥28 ^+0^ weeks).

•    Preterm birth rate_e (enhanced analysis: any birth 22 ^+0^- 36 ^+6^ weeks gestation; denominator total births ≥22 ^+0^ weeks).

Secondary outcomes

•    Early preterm birth rate_m (main analysis: any birth 28 ^+0^ - 31 ^+6^ weeks gestation; denominator total births ≥28 ^+0^ weeks).

•    Early preterm birth rate_e (enhanced analysis: any birth 22 ^+0^ - 31 ^+6^ weeks gestation; denominator total births ≥22 ^+0^ weeks).

•    Extreme preterm birth rate_e (enhanced analysis: any birth 22 ^+0^ - 27 ^+6^ weeks gestation; denominator total births ≥22 ^+0^ weeks).

•    Spontaneous preterm birth rate_e (enhanced analysis: any birth 28 ^+0^- 36 ^+6^ weeks gestation which is preceded by spontaneous contractions or preterm prelabour rupture of membranes [PPROM]; denominator total births ≥28 ^+0^ weeks).

•    Spontaneous preterm birth rate_e (enhanced analysis: any birth 22 ^+0^- 36 ^+6^ weeks gestation which is preceded by spontaneous contractions or preterm prelabour rupture of membranes [PPROM]; denominator total births ≥22 ^+0^ weeks).

•    Post term birth rate_m (main analysis: any birth ≥42 ^+0^ weeks gestation; denominator total births ≥28 ^+0^ weeks).

•    Stillbirth rate_m (main analysis: any stillbirth ≥28 ^+0^ weeks gestation (or ≥1000g if gestation not available); denominator total births ≥28 ^+0^ weeks (or ≥1000g if gestation not available).

•    Stillbirth rate_e (enhanced analysis: any stillbirth ≥22 ^+0^ weeks gestation (or ≥500g if gestation not available); denominator total births ≥22 ^+0^ weeks (or ≥500g if gestation not available).

•    Low birth weight rate_m (main analysis: any birth 1000–2500g; denominator live births ≥1000g).

•    Low birth weight rate_e (enhanced analysis: any birth 500–2500g; denominator live births ≥500g).

•    Very low birth weight rate_m (main analysis: any birth 1000 – 1500g; denominator live births ≥1000g).

•    Very low birth weight rate_e (enhanced analysis: any birth 500 – 1500g; denominator live births ≥500g).

•    Extremely low birth weight rate_e (enhanced analysis: any birth 500g – 1000g; denominator live births ≥500g).

### Potential confounders/effect modifiers

Potential confounders/effect modifiers for the entire iPOP study are represented in a DAG ( Figure 2). We recognise that i) many of the variables in the DAG (e.g. maternal age distribution) are unlikely to have significantly changed within the timeframe of the analysis and thus unlikely to be confounders, and ii) our initial analysis strategy is to compare changes in association with lockdown within datasets; thus these variables are less relevant. To allow expedient provision and analysis of data we propose using aggregate data for WP1 and WP2; with more complex analysis enabled with provision of individual participant data and provider level data in subsequent WPs.

National/regional level societal characteristics that we are interested in exploring include mediating and moderating factors obtained from publicly available datasets as described in the section below. Country classification by income as defined by the World Bank (LIC, LMIC, UMIC, HIC) as a proxy for wider social security and healthcare system.

### Data collection and characteristics of datasets

**Figure 4.**
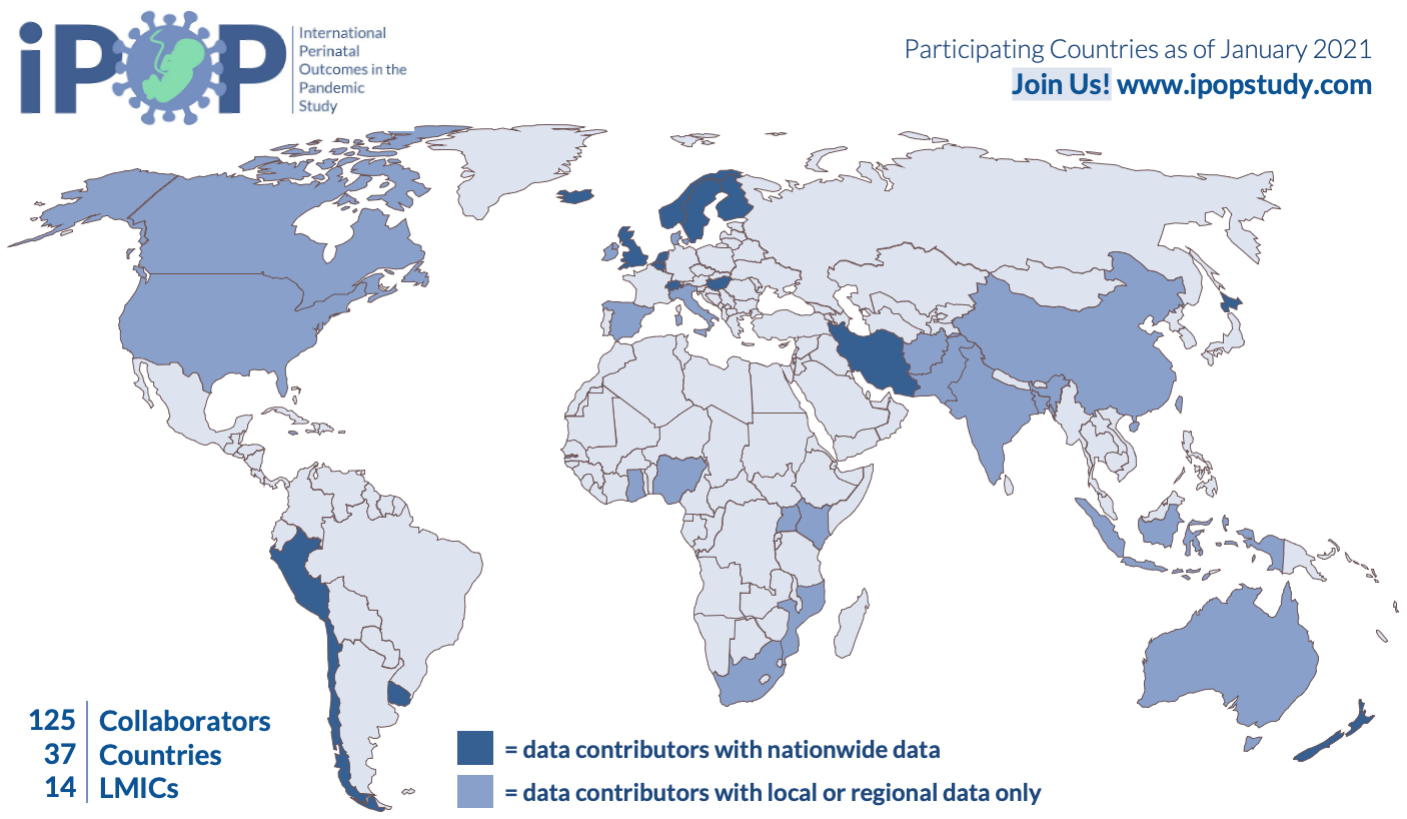
Map of iPOP collaborating countries as of Dec 1, 2020.

We have extended invitations for national, regional and institutional data custodians of birth data to participate through formal and informal networks, social media, lay and scientific media. Participating countries as of December 1 ^st^ 2020 are shown in Figure 4.

We will request aggregate data from each data provider using an excel spreadsheet template, which includes details on levels of missing data. We will classify data provided to iPOP as Standard, Enhanced, or Investigative, based on the characteristics described in Table 1.

**Figure 5.**
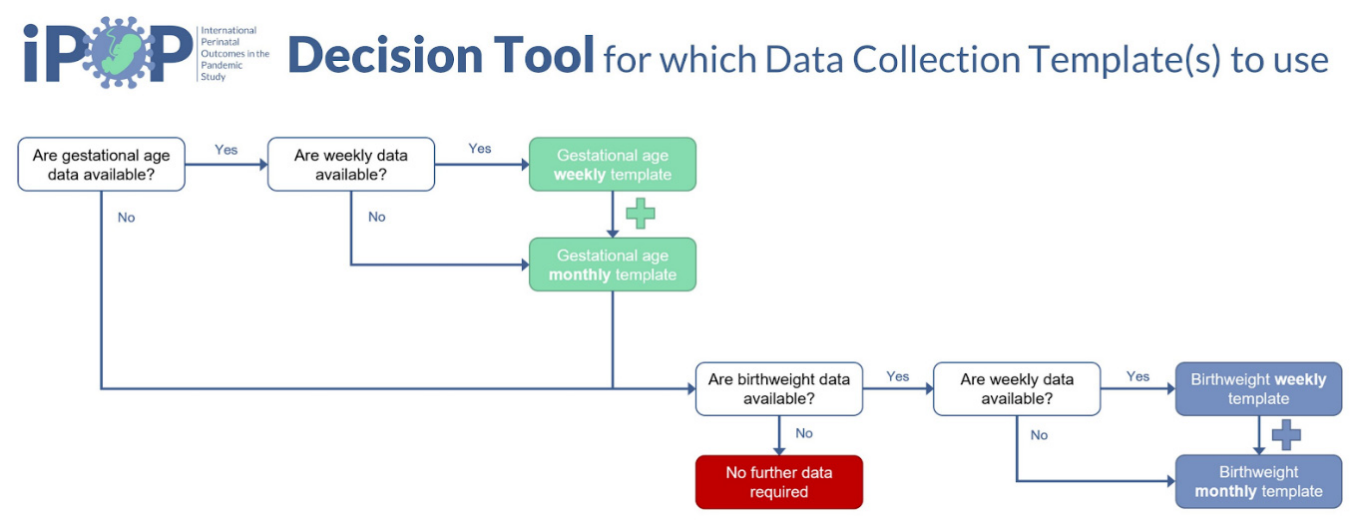
Data request flow diagram.

We will also ask for completion of a questionnaire regarding the source of data including, country of origin, region(s) covered and size of population covered. To assist data providers on which template to use to capture their data, we have constructed a data flow diagram ( Figure 5).

For WP2 we will use the following publicly available data sources:

• *Lockdown stringency:* Using the stringency index (see section *Exposures*) and COVID-19: Containment and Health Index defined as a continuous (0–100) or categorical measures.

• *Socioeconomic status:* Measured by Organisation for Economic Co-operation and Development (OECD) better life index.

• *Ambient air quality:* Estimated using the Data Integration Model for Air Quality (DIMAQ) ^24^, which uses input data from a variety of public sources including: Open Air Quality, NASA Modern-Era Retrospective analysis for Research and Applications version 2 ( MERRA-2) global modelling initiative, satellite imagery data from the Multiangle Implementation of Atmospheric Correction ( MAIAC), and global population density from the NASA/Columbia University Socioeconomic Data and Applications Center.

• *Adherence to lockdown indicated by traffic and movement trends:* Obtained from publicly available Google mobility data.

• *COVID-19 rates:* Nationally available via John Hopkins COVID-19 infection rates

• *Parental leave policy:* Measured by World Bank Data (yes/no; length of paid maternity leave).

• *Other country-level characteristics:* Measured by World Bank Data (including variables such as world region, GDP, income expenditure, hospital beds, maternal education), The Global Gender Gap Index, The Global Hunger Index and Political stability index.

### Data storage and analysis platform

We will use the Secure Anonymised Information Linkage (SAIL) Databank, Swansea Wales, to store all data provided to iPOP. Upon completion of a Data Contribution Agreement between each iPOP data provider and the SAIL Databank, each data providers will either:

i) upload aggregated data directly to the SAIL central repository, or
ii) transfer their data to the University of Edinburgh (RM), who will upload these to SAIL on their behalf.

Data will be transferred into SAIL using the “Split-file” process with the support of the Informatics Service, National Health Services (NHS) of Wales. Person-level demographics are translated to an Anonymous Linking Field (ALF). Additional information on the SAIL File Structure & Data Transfer processes can be found here.

iPOP Team Members (analysis team) will access data stored within SAIL via a remote access and conduct data analyses remotely on the International COVID-19 Data Alliance (ICODA) Workbench, via a federated approach. ICODA is a new data platform that allows scientists and researchers across the globe to discover, access and analyse multi-dimensional datasets in a confidential and secure environment. More information can be found on the HDR UK website.

To ensure outputs are confidential and safe, all statistical outputs will be checked using Statistical Disclosure Control (SDC) procedures before being exported out of the virtual environment. We will use SDC guiding principles from the Handbook on SDC for Outputs by the UK Data Service. This will prevent the identity of a birth from being revealed or inferred from outputs.

A catalogue on the data variables captured will be recorded alongside relevant metadata. These high-level summaries will be made publicly available.

### Data analysis

All analyses will be fully specified in a comprehensive Statistical Analysis Plan. We will adhere to relevant reporting guidance for example the Strengthening the Reporting of Observational studies in Epidemiology ( STROBE).

Descriptive analysis

We will use summary statistics and data visualisations to describe, explore and compare the national/regional data to describe the study outcomes and other perinatal characteristics, including: 

•    All births

•    Live and stillbirths

•    Preterm and post term births

•    Low birth weight

•    Spontaneous preterm births

In WP2 we will use summary statistics and data visualisations (e.g. choropleth maps) to describe, explore and compare the national/regional data.

Statistical modelling

We will undertake population-based ITSA for main analyses of primary and secondary outcomes. We will use time-series techniques to capture any underlying temporal trends and seasonality in the data before the implementation of lockdown measures. We will consider both linear and more flexible trends. We will use these time-series regression models to forecast (or predict) the expected trends and will compare these to the observed trends seen after the lockdown measures. This will capture both immediate (i.e. step) changes and gradual (i.e. slope) changes in the outcome in relation to implementation of lockdown measures in our models. All analyses will be prespecified in a Statistical Analyses Plan before analysis.

Meta-analysis

We will undertake a meta-analysis of national/regional results, on the step-change and the difference between the forecast and observed outcomes at different time points after the implementation of lockdown measures. We will also stratify by country income setting as a dichotomous variable (LIC+LMIC vs UMIC+HIC), since existing data suggests differing effects in these groups. Statistical heterogeneity will be assessed using I ^2^ test.

For WP2, we will use these pooled estimates from WP1 in meta-regression analyses. These will incorporate the moderator/mediator variables as potential mechanisms at a national/regional level. This will measure the influence of these mechanisms on the association between lockdown measures and adverse perinatal outcomes.

Sensitivity analyses

Where enhanced datasets are available for an outcome, we will perform similar modelling techniques to those described above with these enhanced data as sensitivity analyses to test the robustness of the main analyses in different populations. These analyses will be specified further in a comprehensive Statistical Analysis Plan. Predefined examples include:

•    Sensitivity analyses restricting the denominator for our main outcomes of interest (excluding outcomes on spontaneous preterm birth) from all births to only live births. These analyses will be informative for the appropriateness of using datasets which only include information on live births.

•    Sensitivity analyses with varying cut-off points for our lockdown definition (i.e. above and below 50) from the stringency index to test the robustness of assigning ≥50 as the primary cut-off point. These analyses will also allow inclusion of countries with less strict lockdown measures, such as Sweden, and inform whether/to what extent the observed associations might vary by lockdown stringency.

We will conduct supplementary analyses in investigative datasets.

Output confidentiality

All outputs will be checked for any potential disclosure and confidentiality breaches, using guidance from the Handbook on SDC for Outputs by the UK Data Service.

### Public and patient involvement

Public and patient involvement early in study design and development ensures research studies are responsive to input, guidance and advice, and can help identify and mitigate potential challenges early in the research process ^25^. Further, public and patient involvement helps to identify research outcomes that are meaningful and pragmatic to knowledge users.

The iPOP team has engaged parents as patient partners early in the study design and have built a working group to capture and integrate patient involvement in the iPOP study as it moves forward. Meeting monthly, patient partners will be involved in developing effective and meaningful knowledge translation and communication strategies for disseminating iPOP findings. Specific to WP2, patient partners will work with researchers to examine mechanistic effects of the pandemic lockdown on perinatal outcomes. Patient partners will also work with researchers to develop knowledge translation strategies to ensure effective and meaningful dissemination of findings to knowledge users.

### Ethical considerations

To ensure transparent, equitable, and meaningful engagement, we have developed Guiding Principles that outline the terms of agreement for study leads and collaborators who are involved in the iPOP Study. Each member of the iPOP Study must read and sign the guiding principles document in order to collaborate on the study. While not legally binding, this document provides guidance and parameters around authorship, roles and responsibilities, research integrity, communication and Team Science guidelines.

The iPOP Study ensures confidentiality and security of the processing of data for electronic files. Data will be safeguarded by an appropriate level of security, technical and organisational measures to prevent unauthorized disclosure or access, accidental or unlawful destruction, accidental loss or alteration, and unlawful forms of processing. WP1 and WP2 will be based on de-identified aggregate data only.

It will be assumed that any Team member sharing data within the iPOP Study does so in accordance with relevant and applicable legal and regulatory standards and obligations including but not limited to, confidentiality, data protection and intellectual property, and access governance agreements. iPOP collaborators must adhere to these policies and processes.

All collaborators must respect the iPOP principles of data protection and processing, which include the following:

All contributed to iPOP data must be

•    Processed fairly and lawfully

•    Collected for specified and legitimate purposes

•    Accurate

•    Absent of personal identifiers (names, addresses, etc.)

•    Stored not longer than necessary

•    Processed under the responsibility and liability of the data Controller for the provided data set

•    Handled according to the EU GDPR rules (when hosted in the UK)

## Conclusions

Spanning 37 countries ( Figure 4), the iPOP Study brings together expertise in perinatology, epidemiology, environmental science, intersectional feminism, and data science within a collaborative, equitable and interdisciplinary framework. The iPOP Study will leverage the natural experiment arising from the COVID-19 pandemic, to understand possible mechanisms of adverse perinatal outcomes and inform interventions and policy. Further, iPOP will investigate the effects of pandemic lockdowns by country income setting, incorporating data from LICs to HICs across the globe on key perinatal outcomes.

The initial focus of iPOP will be on the impact of COVID‐19 pandemic lockdowns on perinatal outcomes, including preterm birth, low birth weight, and stillbirth. Determining the worldwide extent of changes in perinatal outcomes during the COVID-19 pandemic will advance current understanding of preventable causes of these pervasive perinatal outcomes.

Building on the first two phases of the iPOP Study described in this protocol, iPOP further aims to investigate mechanisms for any observed changes in perinatal outcomes during the COVID-19 pandemic, using individual-level and setting-specific data. In the next study phase (WP3), we aim to examine the impact of maternal comorbidities (e.g. pregnancy complications; pre-existing chronic conditions including mental health), COVID-19 and non-COVID-19 infections, socio-economic factors, prenatal care, and birth practices on any associations between pandemic lockdowns and perinatal outcomes.

Results of the iPOP Study will be rapidly translated through our network of local and international stakeholders to inform further research and testable interventions for improving perinatal healthcare and social support systems during (and well beyond) the COVID-19 pandemic.

## Data availability

No data are associated with this article.
