# Supplementary material for: The international Perinatal Outcomes in the Pandemic (iPOP) study: protocol
Source: Wellcome Open Res. 2021 Feb 2;6:21. [Version 1] doi: 10.12688/wellcomeopenres.16507.1 (PMC8524299; doi:10.12688/wellcomeopenres.16507.1)
Supplement: Peer review history

# Peer review: Reviewer response for version 1

**DOI:** 10.21956/wellcomeopenres.18180.r46152

**Published:** 2021-10-18

**Authors:** Jenny Carter

**Affiliations:** 1 Department of Women and Children’s Health, School of Life Course Sciences, Faculty of Life Sciences and Medicine, King’s College London, London, UK; 2 Guy's and St Thomas' NHS Foundation Trust, London, UK

This paper describes an important and ambitious global study investigating how the COVID pandemic and lockdowns may have affected rates of preterm birth, stillbirth and low birth weight.  The vast multidisciplinary team of 121 researchers in 37 countries have come together to examine variations in COVID-19 rates and lockdown stringency, as well as other factors such as pollution levels, social deprivation and access to maternity care. Findings from the study may lead to a deeper understanding of these factors and what might be done to improve care and outcomes in the future.

 The team’s hypothesis is that the rate of spontaneous preterm birth, low birth weight and/or stillbirth is changed during lockdowns, and that the magnitude and/or direction of change varies between low, low-middle, upper-middle and high income countries.

 The research aims and questions are spelt out clearly and there is detailed explanation of how each dataset will be managed. The figures make a complex protocol easier to understand.

 The authors acknowledge some significant challenges, including the gathering of unreliable and incomplete data and propose methods to account for this, along with the use of internationally recognised definitions. I am not, however, completely convinced it will be possible to unpick spontaneous preterm birth from all preterm births, as I know how unreliable the UK data relating to this can be.

 This is an important and very interesting project; I wish the team all the best and look forward to reading the findings.

Is the study design appropriate for the research question?

Yes

Is the rationale for, and objectives of, the study clearly described?

Yes

Are sufficient details of the methods provided to allow replication by others?

Yes

Are the datasets clearly presented in a useable and accessible format?

Not applicable

Reviewer Expertise:

preterm birth, health data science, risk assessment

I confirm that I have read this submission and believe that I have an appropriate level of expertise to confirm that it is of an acceptable scientific standard.

# Peer review: Reviewer response for version 1

**DOI:** 10.21956/wellcomeopenres.18180.r43887

**Published:** 2021-06-02

**Authors:** Julia Johnson

**Affiliations:** 1 Division of Neonatology, Department of Pediatrics, Johns Hopkins Hospital, Baltimore, Maryland, USA

Thank you for the opportunity to review this study protocol. The investigators propose a 5-year multi-country study to assess the impact of the COVID-19 pandemic on perinatal outcomes, including rates of preterm birth, low birth weight, and stillbirths, using an interrupted time series analysis approach. The study design is appropriate for the described research questions, and the investigators clearly articulate their intended methodological approach. It may be difficult to assess the impact of the implementation of COVID-19 related lockdowns versus the impact of the pandemic (and infection in pregnancy) itself. I agree with the investigators’ decision to focus on gestational age 28+ weeks given the heterogeneity of international data and approaches to classifying viability; the impact of the pandemic/lockdowns on earlier gestational ages will be assessed in a separate analysis (enhanced analysis as described). Classifying stillbirth may be challenging, given the differences in definitions and reporting internationally.

 Minor additional comments:

- Please standardize use of either “health care” or “healthcare” – both terms are currently used in the manuscript.
- Last paragraph of introduction (page 5): Please delete comma after “study” in line 3 of the paragraph.
- Last paragraph of introduction (page 5): Please change “COVID 19” to “COVID-19” in second to last line of the paragraph.
- Figure 1 (page 6): Change “birthweight” to “birth weight” to match remainder of manuscript.
- Figure 2 (page 6): Change “birthweight” to “birth weight” to match remainder of manuscript. Font size is quite small, making DAG difficult to read. If possible, resize figure to fit width of page and increase font size. Current DAG only lists limited number of confounders, though many more could be explored.
- Figure 3 (page 3): Stillbirths are by definition always antenatal – consider using “antepartum” to distinguish from “intrapartum” instead. Strict classification of ante- versus intrapartum stillbirth may be difficult, depending on data sources.
- Outcomes (page 10): Current birth weight categories and numerators/denominators do not quite make sense. Would follow standard WHO or VON categories for defining and calculating these. What about neonates with birth weight less than 500g? Growth-restricted neonates may weigh less than 500 grams but still survive.
- Figure 5: Change “birthweight” to “birth weight” to match remainder of manuscript.

Is the study design appropriate for the research question?

Yes

Is the rationale for, and objectives of, the study clearly described?

Yes

Are sufficient details of the methods provided to allow replication by others?

Yes

Are the datasets clearly presented in a useable and accessible format?

Not applicable

Reviewer Expertise:

Neonatology, global health, infection prevention.

I confirm that I have read this submission and believe that I have an appropriate level of expertise to confirm that it is of an acceptable scientific standard.
